# German version of the engagement of a person with dementia scale: translation and initial application experiences

**DOI:** 10.1007/s00391-024-02346-9

**Published:** 2024-08-23

**Authors:** Kathrin Seibert, Dominik Domhoff, Jacob Fricke, Karin Wolf-Ostermann

**Affiliations:** 1 Faculty 11: Human and Health Sciences, Institute of Public Health and Nursing Research, University of Bremen, Grazer Str. 4, 28359 Bremen, Germany; 2 High Profile Area Health Sciences, University of Bremen, Bremen, Germany

**Keywords:** Dementia, Engagement, Assessment, Psychosocial interventions, Translation, Demenz, Beteiligung, Assessment, Psychosoziale Interventionen, Übersetzung

## Abstract

**Background:**

Assessment tools for engagement in people with dementia often rely on self-reported measures which restricts their use in people with severe cognitive limitations. The Engagement of a Person with Dementia Scale (EPWDS) is a valid and reliable tool to assess behavioral and emotional expressions and responses of engagement in people with dementia through observation; however, the EPWDS is not yet available in the German language.

**Objectives:**

1) Translation and cross-culturally adaptation of the original English version of the EPWDS into the German language (EPWDS-GER) and 2) to gain insights into assessing data with the newly developed instrument.

**Material and methods:**

International recommendations were followed to cross-culturally adapt the English original version of the EPWDS into the German language in 5 steps: translation by three independent translators, synthesis, back translation, expert committee review (*N* = 10) and test of the prefinal version in nursing practice (*N* = 22) on a 5-point Likert scale to assess comprehensibility, practicability and suitability of the EPWDS-GER.

**Results:**

The EPWDS-GER achieved high ratings for the five subscales on ease of understanding, ease of answering and importance of single items for assessing engagement. Average agreement for all items ranged from 3.86 to 4.43 (SD = 0.68–1.29). Overall rating of EPWDS-GER resulted in a mean agreement of 4.18 (SD = 0.73) for suitability and of 4.09 (SD = 0.81) for practicability.

**Conclusion:**

The EPWDS-GER is an easy to use tool for measuring behavioral and emotional expressions and responses of engagement of a person with dementia and can now be utilized in clinical practice and research.

**Supplementary Information:**

The online version of this article (10.1007/s00391-024-02346-9) contains supplementary material, which is available to authorized users.

## Abstract (de)

**Hintergrund:**

Instrumente zur Bewertung der sozialen Beteiligung von Menschen mit Demenz beruhen oft auf Selbstauskünften, was ihre Verwendung bei Personen mit schwereren kognitiven Einschränkungen limitiert. Die Engagement of a Person with Dementia Scale (EPWDS) ist ein valides und zuverlässiges Instrument zur Bewertung von Reaktionen, Verhaltens- und Gefühlsäußerungen der Beteiligung bei Menschen mit Demenz durch Beobachtung. Allerdings ist die EPWDS noch nicht in deutscher Sprache verfügbar.

**Ziel der Arbeit:**

1) Übersetzung und kulturübergreifende Anpassung der englischen Originalversion in die deutsche Sprache (EPWDS-GER) und 2) Gewinnung von Erkenntnissen über die Datenerhebung mit der EPWDS-GER.

**Material und Methoden:**

Die Originalversion der EPWDS wurde gemäß internationaler Leitlinien in fünf Schritten in die deutsche Sprache übersetzt: Übersetzung durch drei unabhängige Personen, Synthese, Rückübersetzung, Überprüfung durch ein Expertenkomitee (*N* = 10) und Test der vorläufigen Version in der Pflegepraxis (*N* = 22), um Verständlichkeit, Eignung und Praktikabilität der EPWDS-GER auf einer 5‑stufigen Likert Skala zu beurteilen.

**Ergebnisse:**

Die EPWDS-GER erzielte hohe positive Bewertungen der Subskalen für leichte Verständlichkeit, leichte Beantwortung und gegebene Relevanz einzelner Items. Die mittlere Zustimmung für alle Items reichte von 3,86 bis 4,43 (SD = 0,68–1,29). Die Gesamtbewertung der EPWDS-GER erzielte eine hohe Zustimmung von durchschnittlich 4,18 (SD = 0,73) für Eignung und 4,09 (SD = 0.81) für Praxistauglichkeit.

**Diskussion:**

Die EPWDS-GER ist ein einfach anzuwendendes Instrument zur Messung der Beteiligung von Menschen mit Demenz sowie ihrer Reaktionen. Die EPWDS-GER ist nun in der klinischen Praxis und in der Forschung verfügbar.

## Background

The global number of people living with dementia is expected to rise and the increasing number of people with dementia highlights the necessity for public health planning and policy initiatives to meet their needs [3]. By the end of 2021 a total of 793,461 people were being cared for in German nursing homes [5] of which 69% are considered to have dementia [17]. Dementia typically progresses over several years, leading to a gradual decline in the quality of life for both patients and their families, necessitating extensive medical, nursing, and social support [6, 12]. Nursing home residents with dementia are especially vulnerable to spend the majority of their time alone while only experiencing few opportunities for engagement in meaningful activities [9]. Psychosocial digital interventions (PDI) for people with dementia (PwD), such as serious games in dementia care aim to influence cognition, physical and psychological functioning, well-being and social participation [13]. Studies evaluating the effects of PDI face the challenge of choosing appropriate instruments to measure outcomes that can range from short-term to long-term effects in the abovementioned target dimensions of PDI and often focus on engagement, apathy or interaction of PwD.

Assessment tools for engagement, social activities or apathy in dementia are often limited by the fact that they often rely on self-reported measures which restricts their use in cohorts of PwD with more severe cognitive limitations [9]. Instruments that aim at observing activities in PwD such as the Maastricht Electronic Daily Life Observation Tool (MEDLO-Tool) [4] are either lacking precise scoring instructions or do not incorporate an overall or subscale score that can be used to evaluate effects of PDI. Other instruments, such as the Apathy Evaluation Scale (AES) do not seem to be suitable to evaluate short-term effects, which have been reported more frequently than long-term effects of PDI [11].

In this context, the Engagement of a Person with Dementia Scale (EPWDS) is a promising tool to assesses the behavioral and emotional expressions and responses of engagement by PwD when partaking in PDI [9]. Engagement can be either a social interaction or the participation in an activity which is assessed through behavioral observations [9]. The EPWDS assesses engagement in five areas (affective, visual, verbal, behavioral and social engagement) and was primarily developed for use in research involving PwD across different care settings [9]. The EPWDS is a 10-item observer-rated instrument designed to examine whether an individual with dementia exhibits an emotional or behavioral expression or response of engagement with, in, or following the introduction of a psychosocial activity [9]. For each item standardized instructions and scoring guidelines with detailed rating descriptions (range 0–5) are provided. The minimum recommended duration of an observational period is 10 min and the EPWDS can be used to establish a baseline comparison prior to the introduction of the activity [9]. The total EPWDS score for all items across all dimensions ranges from 10 to 50, with a higher score indicating a higher level of positive engagement while a lower score indicates a higher level of disengagement or negative engagement [9]. Data collection on the responses of PwD using the EPWDS can be conducted using either video recording or natural observations [9]. Psychometric properties of the EPWDS have been tested in a cohort of PwD in long-term care facilities engaging in a PID with the robotic seal PARO, and the EPWDS has shown robust internal consistency, interrater reliability, test-retest reliability and construct validity [9].

An assessment instrument for engagement with PDI such as the EPWDS is currently not available in the German language. As the EPWDS includes standardized written instructions for the raters observing PwD and classifying the level of engagement, a scientifically valid translation according to established criteria is required. Therefore, the first aim of this study was to translate and cross-culturally adapt the original version of the EPWDS into the German language (EPWDS-GER). The second aim was to obtain feedback from nurses working with PwD on the practicability and comprehensibility of instructions for use of the EPWDS-GER.

## Methods

As there is no universally established reporting standard for studies reporting on the cross-cultural adaption of health measures, the procedure followed the reporting structure given in Gordt et al. [8], who reported on the translation and evaluation of the German version of the Community Balance and Mobility Scale.

### Design: cross-cultural adaption of the EPWDS

Authors of the original version authorized the translation of the EPWDS. The cross-cultural adaption of the original English version of the EPWDS into the German language was done by three researchers in the field of nursing and health sciences. All were fluent in both English and German following the Recommendations for the Cross-Cultural Adaption of Health Status Measures [2], which defines five stages for the translation progress:

#### Stage I: initial translation

The original EPWDS was translated from the English language into the German language by three independent German native speakers (two nursing scientists DD, KS and one data analyst, JF). Two translators had basic knowledge about the concept of engagement assessments and considerable theoretical and practical experience in assessments of cognitive and physical assessments with older adults. One translator had no personal occupational relationship with the assessment topic, but basic knowledge on the concept of engagement of PwD. A translation documentation template was provided to each translator that included highlighting challenging phrases or uncertainties and justifying their decisions, as also recommended by the guidelines for the development and criteria for the adoption of health survey instruments developed by the EU-partnership on health statistics [19].

#### Stage II: synthesis

Based on the three independent translated versions and the original EPWDS, a synthesis into one German version was conducted by three translators in an online discussion. A written report documenting the translation process was provided. The next steps were conducted with the synthesized version.

#### Stage III: back translation

To check the validity of the translated version, the synthesized version was back translated from German to English language. This was carried out by an English native speaker working in a department of health, long-term care and pensions, who is also fluent in German and was blinded to the original version of the EPWDS but informed about the concept of engagement.

#### Stage IV: expert committee review

The expert committee (*N* = 10) consisted of the forward and back translators, four additional public health and nursing scientists and two nurses working with PwD. All members had excellent language skills and methodological experience in the field of cognitive and physical assessments with PwD. The committee reviewed and rated all the translations regarding semantic, idiomatic, experiential, and conceptual equivalence and reached consensus on the prefinal German translation of EPWDS-GER.

#### Stage V: test of the prefinal version

This prefinal version was rated by 23 nurses and other health professionals at the Diakoneo nursing home in Nürnberg, Germany. Participants were asked to rate each subscale of the EPWDS-GER on a 5-point Likert scale for whether the subscale is easy to understand, easy to answer and whether the behavior or expression to be observed is an important aspect of engagement for people with dementia. For 15 rating items, a rating of 1 indicated no agreement whatsoever with the rating criteria, a rating of 5 indicated complete agreement. Finally, two further items asked for an overall assessment of the scale with respect to its suitability for practical use and whether the EPWDS-GER is a suitable instrument to assess the engagement of people with dementia.

In addition, 3 nurses observed 5 residents for 10 min each while they took part in different group activities (group breakfast and lunch, and a PDI: Tovertafel®, a virtual reality-based serious game for dementia care [1]) at four points in time according to the EPWDS-GER guidelines and the instructions for the observation with the EPWDS-GER. The EPWDS-GER was converted to an electronic version using LimeSurvey (LimeSurvey GmbH, Hamburg, Germany) and observers used a tablet pc for the assessment. Overall, 48 single assessments with the EPWDS-GER were obtained. The observers were afterwards asked if they understood the instructions and the EPWDS-GER and summarized their experience with utilizing the scale in a written memo.

## Results

The stages I–V of the cross-cultural adaption were performed as described. During the synthesis, non-matching translation terms were discussed among the three translators while considering the original version. Decisions were made together amongst the three translators regarding 1. “engagement” = “Beteiligung”, 2. “disengagement” = “Abwesenheit von Beteiligung”, 3. “person with dementia” = “Person mit Demenz”, 4. “community and long-term care” = “ambulante und stationäre Langzeitpflege”, 5. “start time of observation period” = “Beginn des Beobachtungszeitraums”, 6. “end time of observation period” = “Ende des Beobachtungszeitraums”. Cross-cultural adaption was made for the term “community care” by translation to “ambulante Pflege” as a literal translation does not correspond to the term used for community care in Germany.

Based on the translation and back translation, the prefinal version was discussed in the expert committee. In cooperation with the expert committee, it was decided to translate the terms “individual with dementia” or “person with dementia” as “Person mit Demenz” and “people with dementia” as “Menschen mit Demenz”. Furthermore, it was decided to translate “disengagement” as “fehlende Beteiligung” and “distressed” as “Verzweiflung” as this was considered to be more in line with everyday language and observations in nursing practice. The same applies to “facilitator” which was translated to “Anleiter:in”. The final German version (Appendix 1) is officially released and can be downloaded for free.

### Rating of EPWDS-GER by nurses and other health professionals

A total of 23 nurses and other health professionals provided feedback on comprehensibility, ease of answering and relevance of each subscale and provided an overall rating on general suitability and practicability of the EPWDS-GER. One person did not provide information on their professional background and was therefore excluded from the analysis. Out of the remaining 22 participants, 6 provided incomplete answers resulting in sporadically missing values for single items. The majority of participants were nurses with a working focus on geriatric care (*n* = 17, 77.27%). Two participants had a working focus in social care and support for older people, another two were nursing assistants and one person held a management position in long-term care.

Results from the rating generally showed agreement for the five subscales of the EPWDS-GER on ease of understanding and answering as well as on the importance of single items for the engagement of PwD. The average agreement ranged from 3.86 to 4.43 (standard deviation 0.68–1.29) (Table 1). One participant did not agree on the ease of understanding for the subscale for affective engagement. Otherwise, there were no other participants who selected the least favorable answer option for any of the items assessed.

**Table 1:** Results for rating of EPWDS-GER by nurses and other health professionals (*N* = 22)

	Mean and standard deviation (SD) Response ranges from 1 (strongly disagree) to 5 (strongly agree)					
	Overall, the subscale is easy to understand		Overall, the subscale is easy to answer		For people with dementia, [the responses to an activity included in the subscale] are important aspects of engagement	
Subscale	Mean	SD	Mean	SD	Mean	SD
Affective engagement	3.90^A^	1.29	4.00^A^	1.03	3.95^A^	1.10
Visual engagement	4.09	0.97	4.14	0.99	4.43^B^	0.68
Verbal engagement	4.32	0.84	4.19^B^	0.87	4.24^B^	0.83
Behavioral engagement	4.18	1.01	4.33^B^	1.02	4.32	0.89
Social engagement	3.95	1.00	3.86	1.04	4.14	0.77

^A)^ *N* = 20

^B)^ *N* = 21

An overall rating of the suitability of EPWDS-GER to assess the extent of engagement of PwD during an activity and the practicability of the EPWDS-GER resulted in general favorable agreement with the instrument with a mean of 4.18 (SD = 0.73) for suitability and a mean of 4.09 (SD = 0.81) for practicability. It is notable that, compared with the responses of nurses with a working focus in geriatric care, responses of the two nursing assistants were more likely to result in a lower rating of the ease of understanding and answering the EPWDS-GER and the importance of single items.

Memos from the 48 observations with nursing home residents during group activities and during participation in a PDI revealed that items containing various examples for the behavior or emotional expression to be observed (e.g., disinterest, distressed, restlessness, repetitive rubbing of limbs or torso, repeated movement, frowning, crying, moaning, and/or yelling as examples for expressing negative affect) were more difficult to assess than items targeting a single behavior (e.g., maintaining eye contact with the activity, materials used or the person/s involved). Memos also suggested changing “Person mit Demenz” (person with dementia) to “Person mit dementieller Erkrankung” (person with a dementia disorder), which was decided against based on the expert panel discussion in order to maintain a short wording closer to the everyday language of nurses.

## Discussion

The EPWDS was successfully translated into the German language and evaluated regarding comprehensibility, ease of use and general suitability with nurses and residents in a German nursing home. Overall, the results show that the EPWDS-GER is a feasible, easy to understand and easy to use tool for measuring behavioral and emotional expressions and responses of engagement of persons with dementia. The EPWDS-GER can now be utilized in clinical practice and research on psychosocial (digital) interventions for people with dementia. As the majority of participants reported comprehending the EPWDS-GER instructions and subscales, translation and cross-cultural adaption was successful. In addition, construct validity of the EPWDS-GER was confirmed as the overall rating of the suitability of the EPWDS-GER to assess engagement in people with dementia by nurses and healthcare professionals resulted in a favorable consensus of the instrument. This expert rating also provides an indication of the face validity of the EPWDS-GER, for which the significance as a quality feature of a test is critically discussed in the literature [14]; however, as no changes were made to the core concepts of the EPWDS during the translation and cross-cultural adaptation, the findings from the construction process of the original EPWDS-version can be transferred, which shows the scale to have a high level of content and construct validity [9]. Experiences from the field testing of the EPWDS-GER further indicate that the scale can be used to assess different types of social activities which may or may not include psychosocial digital interventions.

Further experiences with the EPWDS-GER are available, as the scale was used in a longitudinal monocentric within-subject study that aimed to assess the effects of the Tovertafel® in a sample of 25 nursing home residents with moderate to severe dementia who participated in a Tovertafel® group intervention for 8 weeks [10]. The EPWDS-GER was used to assess the level of engagement at three points in time (during group breakfast as a control activity, during the Tovertafel® intervention, 1h after the Tovertafel® intervention) and found positive effects for the PDI intervention in terms of an increase in positive social engagement,(e.g., using the intervention to encourage others to interact or as a communication channel), a decrease in negative social engagement (e.g., distracting or disrupting others) during the PDI intervention, and an increase in positive behavioral engagement (e.g., approaching, reaching out) which was highest 1h after the PDI while negative behavioral engagement (e.g., avoiding, shoving away, pulling back from) decreased [10]. The study demonstrates the applicability of the EPWDS-GER in a real-world research setting in a German population.

## Limitations

By including two informed forward-translators and one uninformed forward-translator the process ensured guideline-compliant conduction of stage I of the translation process but deviated from guidelines in stage II as synthesis was achieved through discussion of the three translators from stage I without including an unbiased additional person [2]. Deviating from recommendations for cross-cultural adaption of health status measures [2], back translation was performed by only one translator who can be considered uninformed. Similar deviations are described by Gordt et al. [8], who should be referred to for reporting purposes, and who point out that the evidence base for the benefit of including a second back translation is relatively weak. For the test of the prefinal version (stage V), guidelines recommend a sample of 30–40 participants [2]. The study did not fully reach this sample size but the sample of 25 nurses and healthcare professionals (rating of the scale and test observations) is considered to be sufficient, taking into account that the survey and assessment took place during enforced measures to prevent the spread of the COVID-19 pandemic in 2021 when access to nursing homes was highly restricted and nursing home staff and residents were exposed to a particular strain [16]. From the latter arises the most major limitation of the results, as the COVID-19 pandemic was the leading cause for nursing homes or geriatric day-care facilities to not participate in a validation study for internal consistency, inter-rater reliability and test-retest reliability for which ethical approval was obtained by the German Society for Nursing Science. In addition, statistical procedures to determine the construct validity of the EPWDS-GER, such as a multitrait-multimethod analysis [15], were not applied as the results from the expert rating in combination with the sound validity of the original version were considered as sufficient. Due to funding restraints, we can only report on translation and cross-cultural adaption, construct validity, and initial field experiences; however, a systematically translated version of the EPWDS in the German language is now available which stands out from ad hoc and on demand translated versions.

## Conclusion

The EPWDS-GER was successfully developed and applied to assess engagement in people with dementia and is now accessible for the use in clinical (nursing) practice and research in Germany. The EPWDS-GER can be utilized in studies aiming to assess and monitor affective, visual, verbal, behavioral and social engagement in people with dementia as well as in the care and nursing process for selecting and guiding psychosocial (digital) interventions to stimulate engagement and interaction. Furthermore, the EPWDS-GER can also be utilized to develop algorithms for a digitally automated recognition of engagement of people with dementia based on artificial intelligence methods for speech, motion and posture detection and analysis which are being applied in the care for people with dementia more and more frequently [18]. The EPWDS-GER may help to raise awareness of the importance of engaging people with dementia in meaningful activities which is of great importance and in line with current guidelines on shaping relationships with and for people with dementia [7].

## Supplementary Information

Appendix 1: Engagement of a Person with Dementia Scale - Deutsche Version (EPWDS-GER)

## Data Availability

Data can be made available upon reasonable request.

## Declarations

The selected experts were considered a nonvulnerable population in the study context and participation was not classified as of particular risk. After receiving written information on the study including statements on data protection and concordance with the European General Data Protection Regulation (EU-GDPR), participants of the expert interviews, online workshop and online survey provided written consent to participation, to analysis of the data obtained and to publication of anonymized results. Due to the short duration of the study, no additional ethics approval was obtained, as available hearing dates did not correspond with the timeframe of the study. Phrasing and content used in the written information and informed consent statements had previously been used in multiple studies of the research group after prior to receiving approval by ethics committees.
