# Supplementary material for: German version of the engagement of a person with dementia scale: translation and initial application experiences
Source: Z Gerontol Geriatr. 2024 Aug 23;58(2):109–14. doi: 10.1007/s00391-024-02346-9 (PMC11870865; doi:10.1007/s00391-024-02346-9)
Supplement: Supplementary file 1 — Appendix 1: Engagement of a Person with Dementia Scale - Deutsche Version (EPWDS-GER) [file 391_2024_2346_MOESM1_ESM.pdf]

ENGAGEMENT OF A PERSON WITH DEMENTIA SCALE - Deutsche Version (EPWDS-GER)

Die "Engagement of a Person with Dementia Scale" (EPWDS) misst die Verhaltens- und Gefühlsäußerungen und Reaktionen von Menschen mit Demenz, wenn ihnen eine psychosoziale Aktivität (d.h. nicht-pharmakologische Intervention) angeboten wird. Die Skala wurde entwickelt, um zu untersuchen, ob eine Person mit Demenz eine emotionale oder verhaltensbezogene Äußerung/Reaktion der Beteiligung mit, während oder nach der Durchführung der Aktivität zeigt.

Um verschiedene Ausdrucksformen von Beteiligung bei einer psychosozialen Aktivität zu erfassen, misst die EPWDS fünf Dimensionen der Beteiligung: affektiv, visuell, verbal, verhaltensbezogen und sozial. Jede Dimension der Beteiligung sollte einzeln erfasst, aber gemeinsam mit allen Dimensionen interpretiert werden, um einen umfassenden Überblick über die erlebte Beteiligung einer Person mit Demenz gegenüber dem Stimulus zu erhalten.

Jede Dimension besteht aus einer Subskala, die positive Beteiligung misst, und einer Subskala, die fehlende Beteiligung oder negative Beteiligung misst. Die EPWDS-GER erkennt an, dass nicht alle psychosozialen Aktivitäten die fünf Dimensionen der Beteiligung einbeziehen, eine geringere Punktzahl einer bestimmten Dimension der Beteiligung kann jedoch auf eine Einschränkung der psychosozialen Aktivität für die beurteilte Person mit Demenz hinweisen.

Verwendung der Skala

- Die EPWDS wurde in erster Linie für die Forschung mit Menschen mit Demenz in verschiedenen Settings (z.B. Akutpflege, ambulante oder stationäre Langzeitpflege) entwickelt.
- Es wird empfohlen, die EPWDS-GER für Beobachtungszeiträume mit einer Beobachtungsdauer von mindestens 10 Minuten einzusetzen.
- Die EPWDS-GER kann verwendet werden, um einen Vergleichswert vor Beginn der psychosozialen Aktivität zu ermitteln.
- Jedes Item wird auf einer Likert-Skala von 1 bis 5 gemessen. Bitte kreuzen Sie für jedes der Items einen Wert an.
- Die Antwortmöglichkeit "nicht anwendbar" sollte nur dann verwendet werden, wenn eine bestimmte Art der Beteiligung für die Person mit Demenz irrelevant ist oder nicht ermittelt werden kann (z. B. eine Person, die nach einem Schlaganfall ihre Sprachfähigkeit verloren hat).

Auswertung der Skala

- Item 2, 4, 6, 8 und 10 sind umgekehrt bewertete Items. Nach Bewertung des Beobachtungszeitraumes mit der EPWDS-GER drehen Sie einfach die numerische Bewertung der Items 2, 4, 6, 8 und 10 um. Das bedeutet, dass eine Punktzahl von 5 zu 1 wird, 4 zu 2, 3 bleibt 3, 2 wird 4 und 1 wird 5.
- Nach dem Umdrehen der Bewertung der Items 2, 4, 6, 8 und 10, summieren Sie die Punktzahlen aller 10 Items um ein Gesamtergebnis für die Beteiligung der Person mit Demenz zu erhalten.
- Werden alle Items in allen fünf Dimensionen der EPWDS-GER gemessen, liegt der Gesamtwert im Bereich von 10 bis 50. Je höher der Gesamtwert ist, desto höher ist der Grad der positiven Beteiligung der Person mit Demenz. Je niedriger der Gesamtwert ist, desto höher ist der Grad der fehlenden Beteiligung oder negativer Beteiligung, den die Person mit Demenz zeigt.
- Zur Untersuchung von und Kontrolle auf Umgebungsfaktoren auf den Grad der Beteiligung des Menschen mit Demenz kann eine Interkorrelationsanalyse zwischen der EPWDS-GER-Gesamtpunktzahl und der Bewertung der Umgebungsfaktoren im Abschnitt „Angaben zu Beobachtungszeitraum und psychosozialer Aktivität“ durchgeführt werden.

Angaben zu Beobachtungszeitraum und psychosozialer Aktivität

Beginn des Beobachtungszeitraumes: ___________ Ende des Beobachtungszeitraumes: ___________

Gesamtdauer des Beobachtungszeitraums: ____

Art der psychosozialen Aktivität: ____

Psychosoziale Gruppen- oder Einzelaktivität: ____

Ort der psychosozialen Aktivität: ____

Eignung der Umgebung: Bitte geben Sie an, inwieweit Sie der folgenden Aussage zustimmen oder widersprechen.

Die Umgebung insgesamt (z.B. Beleuchtung, Lärmpegel, Anwesenheit anderer Personen) ist dazu geeignet, dass die beabsichtigte psychosoziale Aktivität eine positive Beteiligung bei Menschen mit Demenz hervorrufen kann.	1<br>☐<br>stimme überhaupt nicht zu	2<br>☐	3<br>☐	4<br>☐	5<br>☐<br>stimme voll und ganz zu

Affektive Beteiligung

Bitte geben Sie an, inwieweit Sie den folgenden Aussagen zustimmen oder nicht zustimmen:

Die Person mit Demenz …

1.	… zeigt positive Affekte wie Vergnügen, Zufriedenheit oder Begeisterung (z. B. Lächeln, Lachen, Entzücken, Freude, Interesse und/oder Enthusiasmus).	1<br>☐<br>stimme überhaupt nicht zu	2<br>☐	3<br>☐	4<br>☐	5<br>☐<br>stimme voll und ganz zu	N/A<br>☐<br>nicht anwendbar
2.	… zeigt negative Affekte wie Apathie, Wut, Angst, Furcht oder Traurigkeit (z.B. Desinteresse, Verzweiflung, Unruhe, wiederholtes Reiben an Gliedmaßen oder Körper, wiederholte Bewegungen, Stirnrunzeln, Weinen, Stöhnen und/oder Schreien).	1<br>☐<br>stimme überhaupt nicht zu	2<br>☐	3<br>☐	4<br>☐	5<br>☐<br>stimme voll und ganz zu	

Visuelle Beteiligung

Bitte geben Sie an, inwieweit Sie den folgenden Aussagen zustimmen oder nicht zustimmen:
Die Person mit Demenz …

		1	2	3	4	5	N/A
3.	… hält Blickkontakt mit der Aktivität, den verwendeten Materialien oder der/den beteiligten Person/en.	☐ stimme überhaupt nicht zu	☐	☐	☐	☐ stimme voll und ganz zu	☐ nicht anwendbar
4	… erscheint unaufmerksam, zeigt ein unfokussiertes Starren oder dreht den Kopf/die Augen von der Aktivität, den verwendeten Materialien oder der/den beteiligten Person/en weg.	☐ stimme überhaupt nicht zu	☐	☐	☐	☐ stimme voll und ganz zu	

Verbale Beteiligung

Bitte geben Sie an, inwieweit Sie den folgenden Aussagen zustimmen oder nicht zustimmen:
Die Person mit Demenz …

		1	2	3	4	5	N/A
5.	… initiiert, beteiligt sich an oder unterhält verbale Konversation, Geräusche oder Gesten (z.B. Nicken) als Reaktion auf die Aktivität, die verwendeten Materialien oder die beteiligten Person/en.	☐ stimme überhaupt nicht zu	☐	☐	☐	☐ stimme voll und ganz zu	☐ nicht anwendbar
6.	… verweigert die Teilnahme an der Aktivität oder an einem Gespräch im Zusammenhang mit der Aktivität durch verbale Äußerungen von z.B. "Nein" oder "Stopp" etc. ODER äußert negative Kommentare, Beschwerden und Geräusche (z.B. Stöhnen oder Schimpfen oder Fluchen) als Antwort auf die Aktivität, die benutzten Materialien oder die beteiligten Person/en.	☐ stimme überhaupt nicht zu	☐	☐	☐	☐ stimme voll und ganz zu	

Durch die Urheberin autorisierte Übersetzung der EPWDS (Originalversion: Jones et al. 2018, https://doi.org/10.1111/jan.13717).
Stand 17.05.2024. Zitation und Kontakt zum Übersetzungsteam: kseibert@uni-bremen.de

Verhaltensbezogene Beteiligung

Bitte geben Sie an, inwieweit Sie den folgenden Aussagen zustimmen oder nicht zustimmen:
Die Person mit Demenz …

		1	2	3	4	5	
7.	… reagiert auf die Aktivität durch Annäherung, Handausstrecken, Berühren, Halten oder Anfassen der Aktivität, der verwendeten Materialien oder der/den beteiligten Person/en.	☐ stimme überhaupt nicht zu	☐	☐	☐	☐ stimme voll und ganz zu	N/A ☐ nicht anwendbar
8.	… reagiert auf die Aktivität durch Vermeiden, Wegschieben, Rückzug, Schlagen oder unsachgemäßen Umgang mit der Aktivität, denn verwendeten Materialen oder der/den beteiligte/n Person/en.	☐ stimme überhaupt nicht zu	☐	☐	☐	☐ stimme voll und ganz zu	

Soziale Beteiligung

Bitte geben Sie an, inwieweit Sie den folgenden Aussagen zustimmen oder nicht zustimmen:
Die Person mit Demenz …

		1	2	3	4	5	
9.	… nutzt die Aktivität oder das/die verwendete/n Material/ien um Andere zu Interaktion zu ermutigen oder als Kommunikationsmittel um mit Anderen (z.B. Personal oder andere Bewohner*innen) zu interagieren und sprechen.	☐ stimme überhaupt nicht zu	☐	☐	☐	☐ stimme voll und ganz zu	N/A ☐ nicht anwendbar
10.	… lenkt als Reaktion auf die Aktivität andere (z.B. Personal/Anleiter*in und andere Bewohner*innen) ab oder stört diese.	☐ stimme überhaupt nicht zu	☐	☐	☐	☐ stimme voll und ganz zu	
